# Does Overloading Cognitive Resources Mimic the Impact of Anxiety on Temporal Cognition?

**DOI:** 10.1037/xlm0000845

**Published:** 2020-05-07

**Authors:** Ioannis Sarigiannidis, Peter A. Kirk, Jonathan P. Roiser, Oliver J. Robinson

**Affiliations:** 1 Institute of Cognitive Neuroscience, University College London; 2 Institute of Cognitive Neuroscience and Department of Experimental Psychology, University College London; 3 Institute of Cognitive Neuroscience and Department of Clinical Education and Health Psychology, University College London

**Keywords:** time perception, attention, working memory load, anxiety, temporal bisection

## Abstract

Anxiety alters how we perceive the world and can alter aspects of cognitive performance. Prominent theories of anxiety suggest that the effect of anxiety on cognition is due to anxious thoughts “overloading” limited cognitive resources, competing with other processes. If this is so, then a cognitive load manipulation should impact performance of a task in the same way as induced anxiety. Thus, we examined the impact of a load manipulation on a time perception task that we have previously shown to be reliably impacted by anxiety. In contrast with our prediction, across 3 studies we found that time perception was insensitive to our load manipulation. Our results do not therefore support the idea that anxiety impacts temporal cognition by overloading limited cognitive resources, at least as induced by a commonly used load manipulation. Thus, anxiety might affect temporal cognition in a unique way, via an evolutionary-preserved defense survival system, as suggested by animal-inspired theories of anxiety, rather than competing for limited attentional resources.

Being in an anxious state can be detrimental to performance of real-life and lab-based tasks (Arnsten, 2009; Robinson, Vytal, Cornwell, & Grillon, 2013). Anxious individuals frequently report that their worrisome thoughts are hard to control, to the point where they may be unable to think of anything else, resulting in interference with everyday tasks. One prominent theory posits that the deleterious effect of anxiety on cognition is because components of anxiety, such as worry and self-preoccupation, take up the limited cognitive processing resources necessary to perform the task at hand, thereby impairing performance (Eysenck, Derakshan, Santos, & Calvo, 2007). An implication of this theory is that the effect of state anxiety on cognition should be similar to that of other manipulations that take up cognitive resources, such as working memory load. Specifically, this account predicts that anxious thoughts should impair performance in a cognitive task the same way that being overloaded by information can impair one’s performance (Sweller, 1988).

Support for the idea of mechanistic similarity between anxiety and working memory load comes from experimental evidence suggesting that they compete for limited attentional recourses. Specifically, it has been found that anxiety impairs working memory, but also the reverse, that anxiety is affected by working memory load. Regarding the former, threat-of-shock induced anxiety (Schmitz & Grillon, 2012) impaired both spatial and verbal working memory (Lavric, Rippon, & Gray, 2003; Shackman et al., 2006). Interestingly, this effect was found under low but not high cognitive load, that is, only when the working memory task was relatively easy (Vytal, Cornwell, Arkin, & Grillon, 2012, 2013). One possibility is that when the working memory task became difficult (i.e., high cognitive load), attentional resources were shifted toward performing the cognitively demanding task and away from the mildly threatening event (i.e., anticipating a shock during the threat-of-shock condition). If this is the case then one would expect that shifting attention away from threat would lead to decreasing anxiety under this high cognitive load condition. Indeed, this has been shown by a study in which anxiety was measured using a physiological marker: threat-potentiated startle (King & Schaefer, 2011). In another study, when participants were under high cognitive load, the physiological measure of anxiety decreased compared with the low cognitive load condition (Balderston et al., 2016). Taken together these studies indicate that both state anxiety and working memory load can compete for limited attentional resources.

Further support for mechanistic similarities between anxiety and working memory comes from studies examining their effects on arousal. A number of threat of shock studies find increased physiological markers of arousal including skin conductance and startle reflex (Bradley, Zlatar, & Lang, 2018) as well as pupil dilation (Bitsios, Szabadi, & Bradshaw, 1996). Similarly, working memory load increases pupil dilation and this effect occurs across a large range of tasks (for a meta-analysis see van der Wel & van Steenbergen, 2018). In the cognitive domain, it has been reported that cognitive load (Brown, 1997) and clinical anxiety (Bar-Haim, Kerem, Lamy, & Zakay, 2010) shift time perception in a similar way, although the timing tasks that were used in these studies were not directly comparable.

Overall, this evidence suggests that working memory load influences arousal in a similar way to anxiety and that both load and anxiety compete for similar attentional resources. However, to our knowledge no study has directly assessed whether working memory load and anxiety impact temporal cognition in the same way on identical cognitive tasks. In this study, we therefore combined a time perception task (Kopec & Brody, 2010) which we have previously shown to be influenced by induced anxiety (Sarigiannidis, Grillon, Ernst, Roiser, & Robinson, 2019), with a cognitive load manipulation. In our previous studies we found that anxiety induced by threat of shock (Schmitz & Grillon, 2012) led to the underestimation of temporal intervals. We argued that when participants were in the anxious state, worrisome thoughts related to the anticipation of the shocks took up limited attentional resources, which led participants to underestimate temporal intervals. In other words, anxiety-related thoughts distracted participants from the temporal cognition task at hand. Distraction from time could lead to “missing ticks from our mental clock” (Coull, Vidal, Nazarian, & Macar, 2004; Macar, Grondin, & Casini, 1994; Thomas & Weaver, 1975). If this is the case, then we would expect that temporal underestimation would also occur when participants are overloaded by cognitive load.

We therefore took a well-established load manipulation—increasing set size on the Sternberg task—and predicted that the higher the cognitive load, the more attentional resources dedicated toward maintaining it in memory, and therefore the greater the temporal underestimation in the timing task. In other words, cognitive load should mimic the effects of threat of shock on temporal estimation (see Figure 1).[Fig-anchor fig1]

## Procedure

### Overview

The study and all procedures were approved by the University College London (UCL) Research Ethics Committee (Project ID Number: 1764/001) and were in accordance with the latest version of the Declaration of Helsinki. Participants were recruited from UCL subject databases. During a single testing session, following written informed consent, participants completed questionnaires assessing their mood and anxiety levels. Subsequently they completed the digit span and finally the temporal bisection task under different cognitive load conditions. We conducted three studies on independent samples (see Table 1), so we provide an outline of common methods before providing specific task information.[Table-anchor tbl1]

### General Methods

#### Participants

A power calculation (G*power Version 3.1.9.2; Faul, Erdfelder, Lang, & Buchner, 2007) determined the sample size of Study 1 based on a meta-analysis of previous studies we conducted using the same temporal cognition task and an anxiety manipulation. The meta-analytic effect size of induced anxiety on temporal cognition (Cohen’s *d*) was *d* = 0.68. In order to be conservative, we decreased this effect size by ∼25% to *d* = 0.49; with 80% power and an alpha of 0.05 (two-tailed), the required sample size was estimated to be 35 participants.

Participants had normal or corrected to normal vision and no present (or past) neurological or psychiatric diagnosis. All provided written informed consent and received compensation for their participation (£7.50 per hour).

#### Apparatus

All experiment material was presented on Windows computers using Cogent, 2000 (www.vislab.ucl.ac.uk/cogent.php; Wellcome Trust Centre for Neuroimaging and Institute of Cognitive Neuroscience, University of College London, London, United Kingdom), running under MATLAB.

#### Self-report 1uestionnaires

Participants completed self-report measures of depression (Beck Depression Inventory [BDI]; Beck & Steer, 1987) and state-trait anxiety (State Trait Anxiety Inventory [STAI]; Spielberger, 1983).

#### Working memory

Working memory was assessed using forward and backward digit span from the WAIS-III Digit Span (Wechsler, 1997). Participants listened to sequences of digits, and then were asked to repeat the sequence back in either forward or reverse order. Sequences were presented in ascending order of difficulty, from two to nine digits (forward) and two to eight digits (backward). The total number of correctly repeated forward and backward sequences were used as a working memory score.

#### Temporal bisection task under load

Participants then completed a visual temporal bisection task under cognitive load (see Figure 2). In the load conditions participants were shown letters to remember while performing the temporal bisection task. In the low load condition there were only two different digits (e.g., SSBBB; Figure 2B), while in the high load condition, all five digits were different (e.g., SXBLP; Figure 2C). Participants also performed the task also under no load, which was used as a baseline. The order of the conditions was counterbalanced across participants. There were 36 trials in each block, and thus a total of six blocks (two for each condition).[Fig-anchor fig2]

A short training phrase preceded the temporal bisection task. It consisted of presenting participants with two anchor durations, a “short” duration (1,400 ms) and a “long” duration (2,600 ms). Each was presented three times, and presentation order was pseudorandomized. In addition, before the beginning of each block (load or no load) the anchor durations were repeated.

Each trial started with a set of letters presented for 1.5 s, which had to be memorized and repeated out loud throughout the trial. A to-be-timed fractal image was then presented for one of six durations: 1,400, 1,640, 1,880, 2,120, 2,360, or 2,600 ms. On each trial participants were required to make a choice: press “short” if the duration of the stimulus was judged to be similar to the “short” anchor, or press “long” if the duration of the stimulus was judged to be similar to the “long” anchor. After the 2-s response limit, a single letter probe appeared on the screen for 2 s. Participants had to indicate with a button press whether that single letter belonged to set shown at the beginning of the trial. The feedback participants received on their performance was “correct,” “wrong,” or “too slow” shown for 500 ms. There was a variable intertrial interval: 1,000 ms, 1,500 ms, or 2,000 ms. Following each block, participants rated how difficult they found the memory task using a visual analogue scale.

The fractal images (12 different images) that participants had to time were pseudorandomized, and presented equally often in each condition to avoid potential biases (Wearden & Ferrara, 1996). The load letters consisted of only consonants that were capitalized in the to-be-remembered, set while the single probe letter was in lowercase to avoid engaging visual recognition memory. In order to avoid repetition effects, the same letters were not shown in three consecutive trials. In the no load condition, participants viewed asterisks instead of a set of letters and were instructed to press a button instead of responding to a probe letter. All other aspects of the no load condition were identical to the load condition. All button presses were counterbalanced across participants.

### Data Analysis

Given that we followed same approach in analyzing our data as (Robinson, Bond, & Roiser, 2015), we quote directly from that text. All data was preprocessed in MATLAB (v. R2015b). Frequentist significance tests were run in SPSS (v. 23, IBM Corp., Armonk, NY) while Bayesian analyses were run in JASP (v. 0.9). Frequentist and Bayesian repeated-measures analysis of variance (ANOVA) models were constructed in exactly the same manner for all analyses, with frequentist ANOVAs used to generate *F*-statistics, *p* values, and effect sizes for interactions of interest, and Bayesian ANOVAs used to generate log Bayes factors (logBF_10_) for models of interest relative to a null model.

The Bayesian approach also allows us to compare the relative predictive ability of a given model. Hence rather than, for example, stating that a main effect of load, main effect of time, and their interaction are significant we could say, on the basis of a model comparison that the interaction (for instance) is actually the more parsimonious model. In the Bayesian analyses, the “winning” model was defined as the model with the highest BF_10_ relative to the null, and the relative predictive success of one model over another was computed by dividing the BF_10_ for one model by the other. Any value greater than one indicates a model better than the comparison. Semantic labels were assigned to the magnitude of these comparisons to aid interpretation, ranging from anecdotal (1–3), to substantial (3–10), to strong (10–30), to very strong (30–100), to decisive (>100; Jeffreys, 1998). Where reported for interactions, the Bayes factors represent a model including the interaction plus the main effect of each component of the interaction.

#### Proportion of long responses

Trials on which participants did not make a response were excluded from the analysis. Repeated-measures analyses of variance (ANOVAs) were performed on the proportion of stimuli participants judged to be long (proportion of long responses, p(long)). The effects of load (no load vs. load), duration (six stimulus durations) were used as within-subject factors. Greenhouse-Geisser corrections were applied when violations of sphericity occurred.

#### Psychophysical modeling

Given that data analysis is similar to that of our previous article, some of the phrasing of this section is identical to Sarigiannidis, Grillon, Ernst, Roiser, and Robinson (2019). For each participant we fitted psychometric functions to trials separately for the different load conditions, and computed the bisection point (BP) and Weber fraction (WF). The BP is the time interval that is perceived to be equidistant between the shortest and longest anchor; that is, the time interval corresponding to 50% pLong (Kingdom & Prins, 2010). It provides a measure of the perceived duration of comparison intervals. A rightward shift of the psychophysical curve would lead to a greater BP, indicating underestimation of time (and vice versa for a leftward shift). The WF is a measure of the precision of sensory discrimination (Kingdom & Prins, 2010). The more sensitive participants are to the task durations, the more quickly the curve will rise at its steepest point. A small WF indicates that small differences between the stimuli are detectable, in other words that sensitivity is higher. Paired samples *t* tests were employed to compare BP and WF across the safe and threat conditions.

The data was modeled using the Palamedes toolbox in MATLAB (Prins & Kingdom, 2009). The proportion of long responses, pLong, at each comparison interval, was fitted with logistic functions defined by four parameters: threshold α, slope β, guess rate γ, and lapse rate λ. In line with previous studies, γ was fixed at 0 because the task was two-alternative forced-choice; λ was fixed at 0.1 to allow for occasional attentional lapses (Terhune, Sullivan, & Simola, 2016). α and β were free parameters and estimated using maximum likelihood estimation. The duration corresponding to the 50% threshold on the psychometric function was defined as the BP. To calculate the WF we calculated the difference between the durations corresponding to the 75% and 25% thresholds, and divided by twice the BP, or *t*(pLong = 0.75) − *t*(pLong = 0.25)/2 × BP, where t is the interval duration at the respective location on the fitted psychometric function.

### Experiment Specific Methods

Following Study 1, we further decreased the initial effect size for the power analysis (*d* = 0.68) by ∼50% to *d* = 0.35; with 80% power and an alpha of 0.05 (two-tailed), the subsequent sample size was estimated to be 66 participants. The low load condition was dropped after Study 1, as it did not differ significantly from baseline. For Study 2, participants repeated letters in their head, because participants were within earshot during testing and we wanted to minimize distraction. Lastly, the high load condition was increased to eight digits for Study 3 (see Table 2).[Table-anchor tbl2]

## Results

### Manipulation Check: Effect of Load

There was a significant main effect of load on accuracy for Study 1, *F*(2, 68) = 35.77, *p* < .001, η_p_^2^ = .513; Study 2, *F*(1, 65) = 84.43, *p* < .001, η_p_^2^ = .565; and Study 3, *F*(1, 66) = 314.75, *p* < .001, η_p_^2^ = .827. Participants were less accurate when they had to identify presence of a letter from: five letters compared with two/no letters (Study 1); five letters compared with no letters (Study 2); eight letters compared with no letters (Study 3; Figure 3). Bayes factor analysis revealed the winning models to be those including a main effect of load for Study 1 (logBF_10_ = 19.69), Study 2 (logBF_10_ = 24.25), and Study 3 (logBF_10_ = 54.40), all of which were decisively (>100 times) better than the null models.[Fig-anchor fig3]

### Study 1: No Load, Low Load, and High Load

#### Proportion of long responses

There was a significant main effect of stimulus duration, *F*(2.13, 72.50) = 164.49, *p* < .001, η_p_^2^ = .829. As expected, the longer the stimulus duration, the more likely it was to be classified as “long” (see Figure 4). Contrary to our hypothesis, however, load did not significantly affect the proportion of “long” responses, *F*(2, 68) = 2.46, *p* = .093, η_p_^2^ = .068. The stimulus duration by load interaction was nonsignificant, *F*(10, 340) = 1.11, *p* = .356, η_p_^2^ = .032.[Fig-anchor fig4]

Bayes factor analysis revealed the winning model to be one including only a main effect of duration (logBF_10_ = 334.15), which was anecdotally (two times) better than the model including duration and load (logBF_10_ = 333.44), decisively (391 times) better than the model additionally including a duration by load interaction (logBF_10_ = 328.18), and decisively (>1000 times) better than the load only model (logBF_10_ = −2.96).

#### Psychophysical modeling

Data from one participant was excluded from this analysis because it was impossible to fit an accurate sigmoid curve to their data.

##### Bisection point

The BP was not significantly different between the conditions, *F*(1.52, 50.22) = 0.44, *p* = .592, η_p_^2^ = .013. Thus, there was no shift in the psychometric curve and therefore the perception of temporal intervals did not differ under load.

Bayes factor analysis favored the null model, which was substantially better (four times) than the model including load (logBF_10_ = −1.5).

##### Weber fraction

WF was not significantly different between the conditions as revealed with a repeated-measures analyses of variance, *F*(2, 66) = 0.27, *p* = .763, η_p_^2^ = .008. Thus, there was no evidence that the sensitivity to time intervals differed under load.

Bayes factor analysis favored the null model, which was substantially better (four times) than the model including load (logBF_10_ = −1.48).

### Study 2: No Load and High Load

#### Proportion of long responses

There was a significant main effect of stimulus duration, *F*(2.55, 166.01) = 406.60, *p* < .001, η_p_^2^ = .862. As expected, the longer the stimulus duration, the more likely it was to be classified as “long” (see Figure 5). Similarly to Study 1, load did not affect participants’ proportion of “long” responses, *F*(1, 65) = 0.54, *p* = .466, η_p_^2^ = .008. The stimulus duration by load interaction was nonsignificant, *F*(4.21, 274.14) = 0.95, *p* = .440, η_p_^2^ = .014.[Fig-anchor fig5]

Bayes factor analysis revealed the winning model to be one including only a main effect of duration (logBF_10_ = 521.73), which was substantially (seven times) better than a model including duration and load (logBF_10_ = 519.75), decisively (884 times) better than the model additionally including a Duration × Load interaction (logBF_10_ = 514.99), and decisively better (>1000) than the load only model (logBF_10_ = −2.43).

#### Psychophysics modeling

Data from two participants was excluded from this analysis because it was impossible to fit an accurate sigmoid curve to their data.

##### Bisection point

The BP was not significantly different during the high load (*M* = 2,104.58, *SD* = 262.85) compared with the no load (*M* = 2,147.30, *SD* = 264.83) condition, *t*(63) = 1.40, *p* = .165, *d* = 0.18. Thus there was no shift in the psychometric curve and hence the perception of temporal intervals did not differ under load.

Bayes factor analysis favored the null model, which was substantially better (3.5 times) than the model including load (logBF_10_ = −1.29).

##### Weber fraction

The WF was not significantly different during the high load (*M* = 0.13, *SD* = 0.08) compared with the no load (*M* = 0.13, *SD* = 0.07) condition, *t*(63) = 0.69, *p* = .494, *d* = 0.08. Thus, there was no evidence that the sensitivity to time intervals differed across load.

Bayes factor analysis favored the null model, which was substantially better (six times) than the model including load (logBF_10_ = −1.91).

### Study 3: No Load and High Load

#### Proportion of long responses

There was a significant main effect of stimulus duration, *F*(2.948,194.582) = 266.62, *p* < .001, η_p_^2^ = .802. As expected, the longer the stimulus duration, the more likely it was to be classified as “long” (see Figure 6). There was no main effect of load on proportion of “long” responses, *F*(1, 66) = 1.523, *p* = .222, η_p_^2^ = .023. The stimulus duration by load interaction was significant, *F*(5, 330) = 3.43, *p* = .005, η_p_^2^ = .049; however, all post hoc paired-samples *t* tests for the duration by load interaction were nonsignificant (*p* > .05), except the longest duration, *t*(66) = 2.693, *p* = .009, which did not survive Bonferroni correction (corrected α < .0083).[Fig-anchor fig6]

Bayes factor analysis revealed the winning model to be one including only a main effect of duration (logBF_10_ = 408.06), which was anecdotally (two times) better than a model including duration and load (logBF_10_ = 407.14), strongly (20 times) better than the model additionally including a Duration × Load interaction (logBF_10_ = 405.12), and decisively better (>1000 times) than the load only model (logBF_10_ = −2.03).

#### Psychophysics modeling

##### Bisection point

The BP was not significantly different during the high load (*M* = 2,172.84, *SD* = 657.12) versus no load (*M* = 2,101.07, *SD* = 479.93) condition, *t*(66) = −1.196, *p* = .236. There was no shift in the psychometric curve and hence the perception of temporal intervals did not differ under load.

Bayes factor analysis favored the null model, which was substantially better (four times) than the model including load (logBF_10_ = −1.33).

##### Weber fraction

The WF was not significantly different during the high load (*M* = 0.13, *SD* = 0.19) compared with the no load (*M* = .03, *SD* = .90) condition, *t*(66) = 1.036, *p* = .304. Thus, there was no evidence that the sensitivity to time intervals differed across load.

Bayes factor analysis favored the null model, which was substantially better (four times) than the model including load (logBF_10_ = −1.50).

#### Moderating effects of anxiety

We have included a supplementary analysis for Study 3 which uses trait-level anxiety as a covariate, to rule out that findings are being masked by individual differences in anxiety levels (i.e., trait-anxiety correlates with susceptibility to load-induced temporal underestimation). These analyses suggest no significant interactions with trait anxiety (see the online supplemental materials).

## Discussion

Our results provide no evidence for the hypothesis that cognitive load impacts time perception in the same way as induced anxiety. Specifically, participants did not underestimate time under high cognitive load (5 & 8 load), while in a previous study participants did underestimate time under induced anxiety (Sarigiannidis et al., 2019). Using psychophysical modeling, we also did not detect a significant effect of cognitive load in the task: Neither the perception of time (BP), nor the sensitivity to the time intervals (WF) was affected. In other words, cognitive load did not lead participants to perceive the time intervals as shorter and did not impair the ability to discriminate between different time intervals. Moreover, Bayesian analyses provided strong evidence for the null hypothesis that the load manipulation we used had no impact on time perception.

Our results do not support the idea that anxiety impacts temporal cognition by overloading limited cognitive resources. Specially, three experiments failed to find that a commonly used manipulation of cognitive load mimics the effect of induced anxiety on a time perception task. Therefore, although cognitive theories of anxiety suggest that anxious thoughts overload limited attentional resources thus leading to cognitive deficits, it might be the case that anxiety impairs temporal cognition in a unique way compared to other processes. That is to say we are not suggesting that anxiety does not occupy cognitive resources, but that depletion of cognitive resources (at least as induced by this load manipulation) is not the mechanism by which anxiety influences time estimation. It should be noted that our prior anxiety effect (Sarigiannidis et al., 2019) was looking at the impact of “adaptive” anxiety induced by threat of unpredictable shock in healthy volunteers. Whether these effects generalize to severe anxiety disorders is up for investigation.

Animal-inspired models of fear and anxiety posit that threat activates both a cognitive circuit which relies on working memory, as well as an evolutionary conserved subcortical defense survival system which alters behavior and physiology to promote harm avoidance (LeDoux & Pine, 2016; Moran, 2016). This latter system might be responsible for the unique effect of anxiety on our time perception task. In order to test this behaviorally, a future study could combine our cognitive task with CO_2_ inhalation (Bailey, Argyropoulos, Kendrick, & Nutt, 2005), a manipulation that strongly induces physiological symptoms of fear and anxiety, thus potentially activating the defense survival system. If this manipulation were to mimic the effect of induced anxiety (i.e., Sarigiannidis et al., 2019), it would suggest that anxiety affects temporal cognition in a unique way, via an evolutionary preserved defense survival system, rather than competing for limited attentional resources. This could be further validated via functional neuroimaging connectivity analysis, to observe whether anxiety’s influence on temporal cognition is mediated by cortical networks recruited for working memory, or through more direct, subcortical projections from anxiety circuity (e.g., amygdala, stria terminalis).

While the focus of the present article is on cognitive load, our hypotheses were motivated by the notion that anxiety induces worrying thoughts, taking up working memory resources (Eysenck et al., 2007) necessary for time perception. Whether or not physiological sensations of anxiety versus worry may have differential impacts on temporal cognition is an interesting question, but we have not attempted to dissociate these two facets of anxiety in our design. Future studies utilizing a cognitive neuroscience approach would be highly suitable for distinguishing these aspects of anxiety. If it is via purely physiological sensations, would this be reflected by the aforementioned subcortical projections? If worry is driving the effect, but not via working memory, what are the cognitive and neural pathways by which it is interfering with time perception processes?

**Figure 1 fig1:**
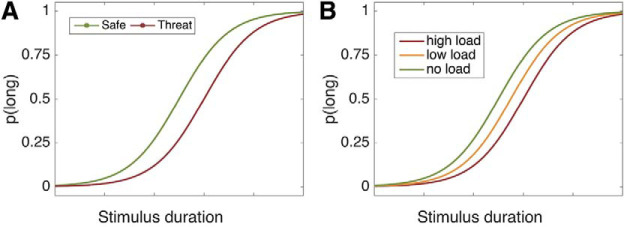
(A) A model of the results from our previous threat of shock study. (B) Predicted effect of working memory load on the time perception task. The curves represent the proportion of long responses, p(long), as a function of stimulus duration and form a characteristic sigmoid shape as stimulus duration increases. We predicted that the cognitive load would promote a rightward shift of the curve, compared to the baseline (no load) condition, due to underestimation of time intervals.

**Figure 2 fig2:**
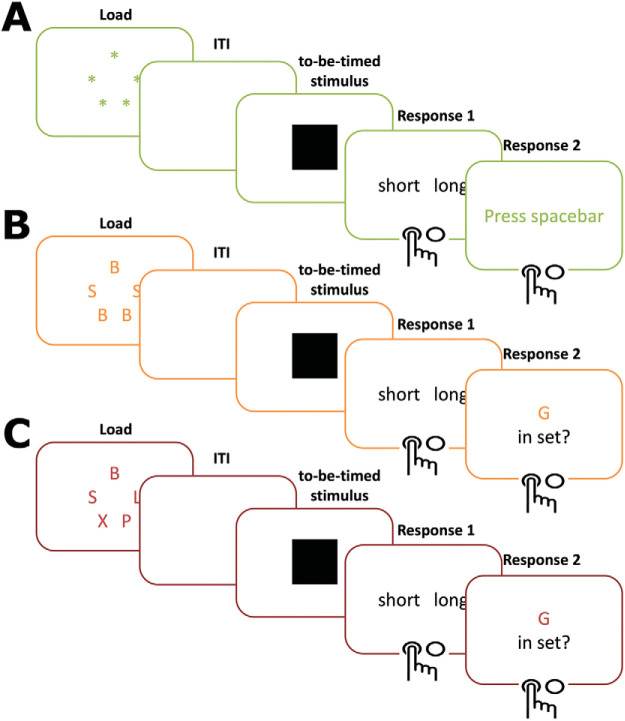
Task design. In Study 1, the task consisted of: (A) no load condition, (B) low load condition, and (C) high load condition. The image colors (green, orange, red) are for illustration purposes and were not used in the actual experiment. In the actual experiment, fractal images were used.

**Figure 3 fig3:**
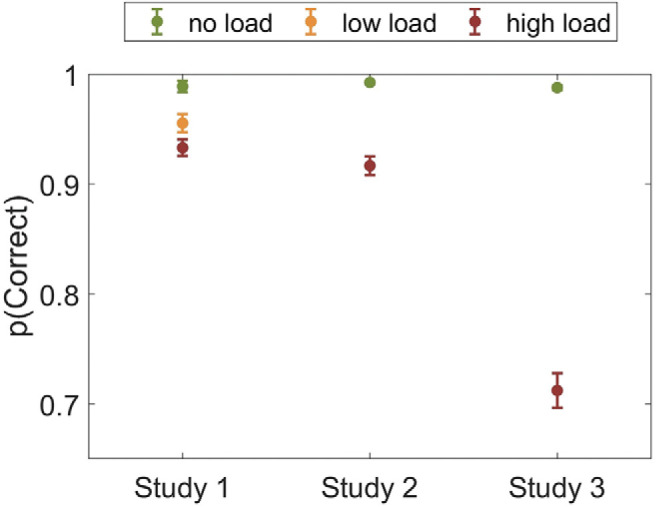
Effect of cognitive load on the load task. Greater values reflect higher accuracy. Error bars are standard errors of the mean (*SEM*).

**Figure 4 fig4:**
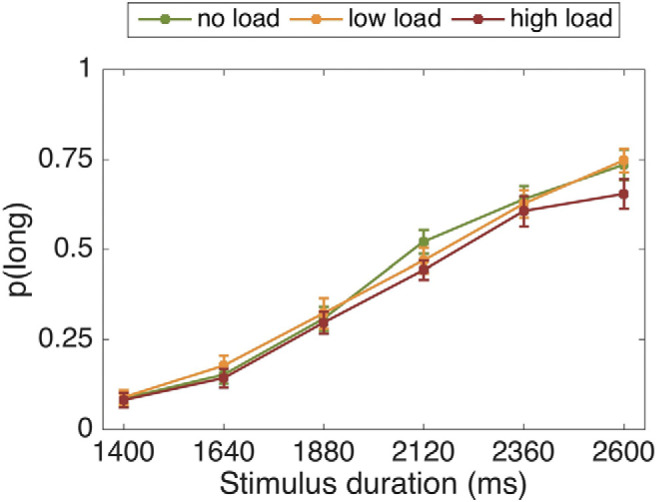
Proportion of stimuli rated “long” as a function of the actual presentation length and load condition. Error bars are standard errors of the mean (*SEM*).

**Figure 5 fig5:**
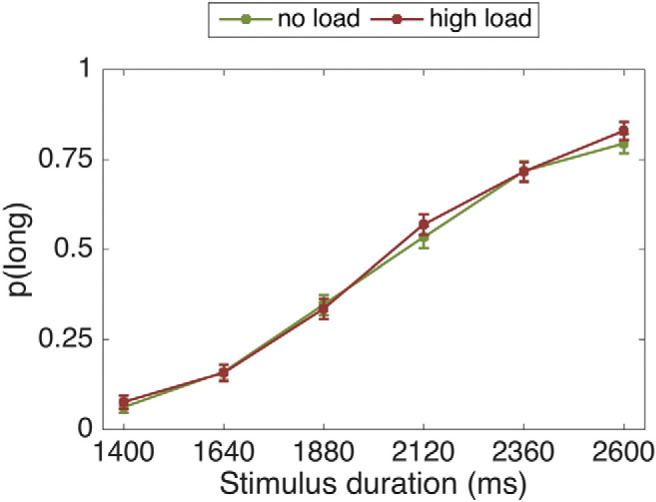
Proportion of stimuli rated “long” as a function of the actual presentation length and load condition. Error bars are standard errors of the mean (*SEM*).

**Figure 6 fig6:**
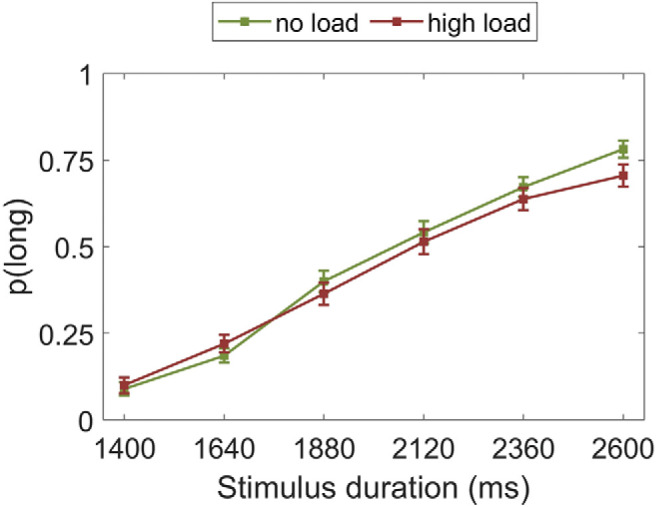
Proportion of stimuli rated “long” as a function of the actual presentation length and load condition. Error bars are standard errors of the mean (*SEM*).

Alternatively, it is possible that anxiety impacts temporal cognition by overloading limited cognitive resources but our load manipulation was not sufficiently taxing to mimic the effect of induced anxiety on the time perception task. In other words, the load task might not have been difficult enough and thus performing it did not result in pronounced competition for attentional resources with the temporal cognition task. In both Experiment 1 and 2, participants’ performance on the high load task was close to ceiling, that is 90%. However, our follow-up Study 3 does not support this. Performance of ∼70% in the high-load (eight characters) condition is closer to chance (50%) than it is full accuracy and yet we still find no evidence for a shifted bisection point or main effect of load comparable with the main effect of threat that we saw previously (Sarigiannidis et al., 2019) and that we predicted a priori (Figure 1B). We did find some weak evidence for a load by duration interaction in the frequentist analysis suggesting that perhaps load does have an effect at longer durations. However, this effect warrants replication, as it does not survive correction for multiple comparisons and neither the Bayesian analysis nor the psychophysiological modeling support it. As such, even if an effect exists it does not clearly match the shift in curves (i.e., significant impact on bisection point) that we observed for threat of shock and predicted a priori.

A further possibility is that we did not have sufficient number of trials to capture effects. However, a previous study of ours made use of just 48 trials per condition (threat-without-shock vs. safe; Sarigiannidis et al., 2019) with 25 participants, which was able to detect an effect. Whereas the current article utilizes 72 trials per condition, with larger sample sizes (35, 66, 67). Therefore, the trial number and sample size are sufficient for capturing similar effects. Lastly, it could be that cognitive load is not a unitary construct and that different cognitive load manipulations (e.g., mental arithmetic) might have effects closer to threat of shock. Future work might seek to test this explicitly.

## Summary

Our results suggest that a commonly used cognitive load manipulation does not affect a time perception task similarly to induced anxiety, as found in our previous work (Sarigiannidis et al., 2019). Thus, these data do not support the proposition that anxiety impacts cognition by overloading limited cognitive resources, because overloading cognition via a load manipulation did not produce the same pattern of results. Instead, our findings are in line with theories of fear and anxiety derived from the animal literature, suggesting that anxiety has a unique effect on behavior and cognition via activating the defense survival system.

## Supplementary Material

10.1037/xlm0000845.supp

## Figures and Tables

**Table 1 tbl1:** Sample Demographic Information for the Three Studies

Study #	Sample size	Age	Female	Digit span	BDI	STAI
Study 1	35	22.71 (2.56)	22	22.11 (2.9)	7.26 (6.60)	40.77 (11.62)
Study 2	66	22.83 (2.66)	46	23.66 (4.3)	N/A	N/A
Study 3	67	23.22 (3.27)	45	17.70 (3.5)	6.46 (5.64)	39.42 (10.34)
*Note*. Figures represent counts or means (*SD*s). BDI = Beck Depression Inventory; STAI = trait anxiety from the State Trait Anxiety Inventory. BDI/STAI measures were not collected for Study 2 due to time constraints.						

**Table 2 tbl2:** Experimental Parameters for the Studies

Study #	Task conditions (digits)	Load digits
Study 1	no load (0), low load (2), high load (5)	repeated out loud
Study 2	no load (0), high load (5)	repeated silently
Study 3	no load (0), high load (8)	repeated out loud
