# Supplementary material for: Does Overloading Cognitive Resources Mimic the Impact of Anxiety on Temporal Cognition?
Source: J Exp Psychol Learn Mem Cogn. 2020 May 7;46(10):1828–35. doi: 10.1037/xlm0000845 (PMC7872305; doi:10.1037/xlm0000845)
Supplement: Supplementary file 1 [file cognitive_load_+_anxiety_supplement_xlm0000845.docx]

**Supplemental Materials**

**Does overloading cognitive resources mimic the impact of anxiety on temporal cognition?**

**by I. Sarigiannidis et al., 2020, *Journal of Experimental Psychology: Learning, Memory, and Cognition***

**Moderating effects of anxiety**

As we measured trait-level anxiety, we decided to supplement our analyses by including this as a covariate of interest, to test whether trait anxiety interacted with our hypothesized effects. Therefore, we carried out repeated-measures ANOVAs on our time perception data for Study 3, but using STAI-measured trait anxiety as a covariate. These analyses suggest no significant interactions with trait anxiety.

### Proportion of long responses

There was a significant main effect of stimulus duration (F(2.929,190.372)=17.30, p<.001, η_p_^2^ =.210). As expected, the longer the stimulus duration, the more likely it was to be classified as “long”. There was no main effect of load on proportion of “long” responses (F(1, 65)=1.173, p=.68, η_p_^2^ =.003). There was no interaction between duration and trait anxiety (F(1,65)=.012, p=.91, η_p_^2^ =.005). The stimulus duration by load interaction was not significant (F(5, 325)=.493, p=.78, η_p_^2^ =.008). There was no interaction between duration, load, and trait anxiety (F(5,325)=.972, p=.435, η_p_^2^ =.015).

Bayes factor analysis revealed the winning model to be one including only a main effect of duration (logBF_10_=408.07). This was: anecdotally (2 times) better than a model including duration and load (logBF_10_=407.15); substantially (<10 times) better than a model including load, duration, and trait anxiety (logBF_10_=405.78); very strongly (20 times) better than a model including a load x duration interaction (logBF_10_=405.063); very strongly (74 times) better than a model including trait anxiety and a load x duration interaction (logBF_10_=403.755); decisively (>1000 times) better than a load only model (logBF_10_=-2.05); and decisively (>1000 times) better than a model including load and trait anxiety (logBF_10_=-3.87).

Psychophysics modelling

*Bisection point*

The BP was not significantly different during the high load (M = 2,172.84, SD = 657.12) vs no load (M = 2,101.07, SD = 479.93) condition (F(1,65)=.494, p=.49, η_p_^2^ =.008). There was BP x trait anxiety interaction (F(1,65)=.174, p=68, η_p_^2^ =.003). Bayes factor analysis favoured the null model, which was anecdotally better (<3 times) than the model including load (logBF_10_=-1.06), and substantially (7 times) better than the model including load and trait anxiety (logBF_10_=-1.88).

*Weber fraction*

The WF was not significantly different during the high load (M = 0.13, SD = 0.19) compared to the no load (M = .03, SD = .90) condition (F(1,65) = .446, p = .51, η_p_^2^ =.007). There was no WF x trait anxiety interaction (F(1,65) = .178, p = .674, η_p_^2^ =.003). Bayes factor analysis favoured the null model, which was substantially better (3 times) than the model including load (logBF_10_=-1.06), and substantially (9 times) better than the model including load and trait anxiety (logBF_10_=-1.88).
